# Searching for solutions to the missing heritability problem

**DOI:** 10.7554/eLife.53018

**Published:** 2019-12-04

**Authors:** Luisa F Pallares

**Affiliations:** 1 Lewis-Sigler Institute for Integrative GenomicsPrinceton UniversityPrincetonUnited States; 2 Department of Ecology and Evolutionary BiologyPrinceton UniversityPrincetonUnited States

**Keywords:** genetic architecture, GWAS, low-frequency variants, complex traits, rare variants, QTL, *S. cerevisiae*

## Abstract

Rare genetic variants in yeast explain a large amount of phenotypic variation in a complex trait like growth.

**Related research article** Bloom JS, Boocock J, Treusch S, Sadhu MJ, Day L, Oates-Barker H, Kruglyak L. 2019. Rare variants contribute disproportionately to quantitative trait variation in yeast. *eLife* **8**:e49212. doi: 10.7554/eLife.49212

**Related research article** Fournier T, Abou Saada O, Hou J, Peter J, Caudal E, Schacherer J. 2019. Extensive impact of low-frequency variants on the phenotypic landscape at population-scale. *eLife* **8**:e49258. doi: 10.7554/eLife.49258

Although most of the 3000 million nucleotides in the human genome are the same in every person on the planet, there are about 90 million sites that can vary between individuals. The source of all phenotypic variation in humans lies in these 90 million genetic variants, and in their interactions with each other and with the environment. Identifying the genetic variants that are involved in a specific trait (such as height or disease status) is a long-standing goal in biology.

Today, researchers rely on genome-wide association studies (GWAS) to find the genetic variants that are relevant to a specific trait. In GWAS the genomes of individuals are analyzed to see if particular genetic variants are correlated with variation in traits of interest. GWAS results have identified hundreds of variants underlying phenotypic variation in humans, mice, fruit flies, rice, maize, and many other taxa. Yet, despite the large number of alleles that have been identified using this technique, the amount of phenotypic variation they explain is just a fraction of what twin and pedigree studies predict is heritable. For example, twin studies have shown that approximately 80% of variation in human height can be explained by genetic factors (Silventoinen et al., 2012). However, the results of the best powered GWAS only explain around 20% of such variation (Wood et al., 2014). This gap is known as the ‘missing heritability problem’.

Rare and low-frequency genetic variants (which have allele frequencies of <1% and <5% respectively) have been proposed as one explanation for the missing heritability problem (reviewed in Gibson, 2012). Such variants are routinely excluded from GWAS studies because when an allele is present in few individuals, the statistical analysis used to draw correlations between traits and alleles is not powerful enough to obtain significant results. As a consequence around 90% of genetic variation in humans and other organisms like yeast has so far gone unexplored (Figure 1A; Auton et al., 2015; Peter et al., 2018). The missing heritability might be hiding in plain sight, but until now, studying the effect of rare alleles on the variation of traits influenced by more than one gene was extremely challenging. Now, in eLife, two independent groups report the results of experiments on yeast which show that rare variants have a fundamental role in phenotypic variation at the population level.

**Figure 1.**
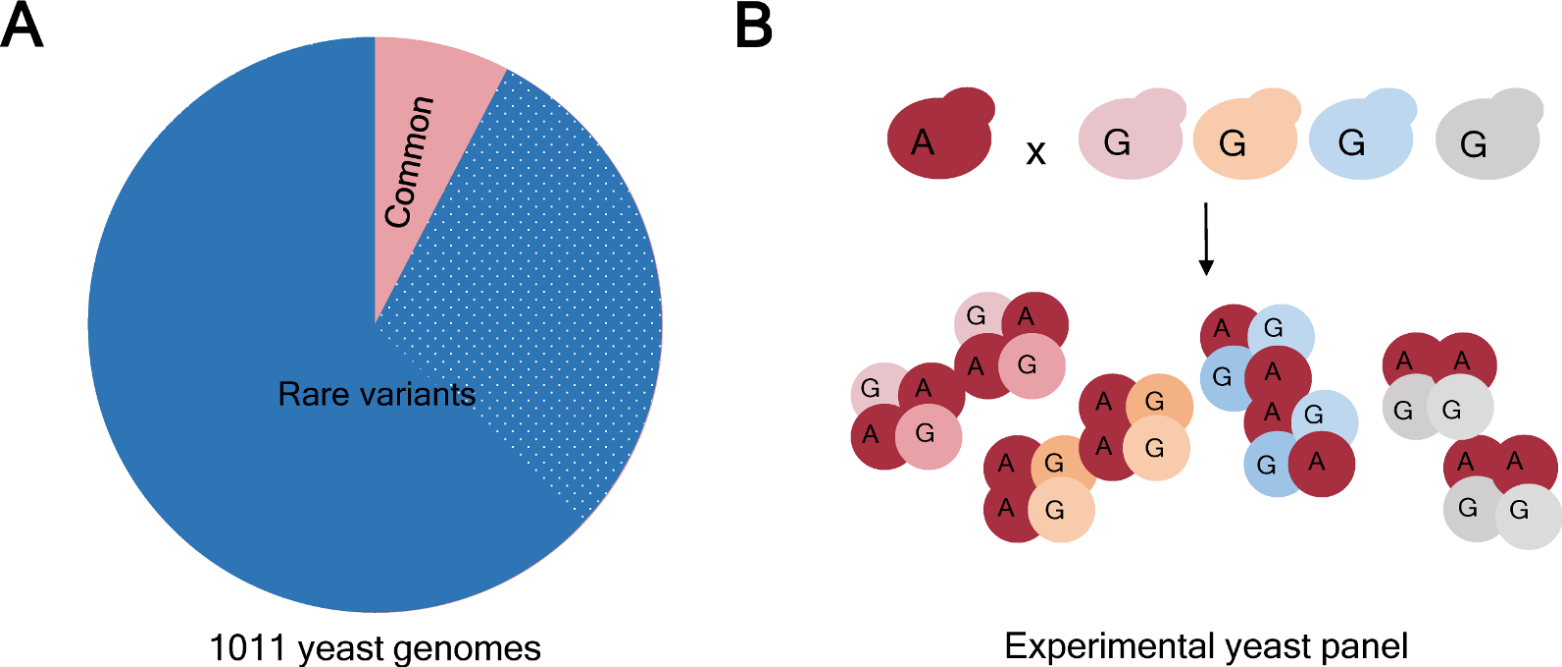
Allele frequency in natural isolates of yeast and in the experimental populations. (**A**) Based on a study of 1011 genomes it is known that 93% of the genetic variants in the yeast *Saccharomyces cerevisiae* are rare (that is, they have a frequency <1%; blue). Moreover, just over 508000 variants (31% of the total; dotted blue) were found in just 1 of the 1011 genomes studied. However, genome-wide association studies (GWAS) tend to focus on the 7% of genetic variants that are common (that is, have a frequency >1%; pink). (**B**) The frequency of a rare allele can be increased by crossing a yeast isolate carrying the rare variant with an isolate with the alternative (more common) variant. To obtain a variety of isolates with a specific rare allele in different genetic backgrounds, the isolate carrying the rare variant (allele A, dark red) can be crossed with several different isolates with the alternative allele (allele G, pink, yellow, blue, grey). As a result, allele A is more frequent in the experimental panel than in the parental isolates, making it suitable for GWAS analysis. Importantly, regardless of the frequency that any allele reaches in the experimental panel, the real natural frequency can be looked up in the collection of 1011 yeast genomes (panel A).

In a monumental effort, the two groups independently selected a set of wild and domesticated yeast isolates from all over the world and crossed them to generate a genetically diverse panel of thousands of strains (Figure 1B). They then exposed each cross to more than 35 different media conditions and quantified their growth by measuring colony size. As a result of the crossing scheme, genetic variants that were present in just one or a few yeast isolates were now present in hundreds of samples in the experimental panels (Figure 1B). This allowed the groups to include a large number of rare variants (up to 28% of the total) in the GWAS analysis: many of these variants would have been excluded from traditional GWAS studies due to their low allele frequency.

Both groups independently identified thousands of genetic variants associated with growth, and estimated that over half of growth variance can be attributed to additive effects. To determine how variants with different frequencies contributed to phenotypic effects, variants were classified into either rare (<1%) and common (>1%) (Bloom et al., 2019), or rare (<1%), low frequency (1–5%) and common (>5%) (Fournier et al., 2019). This classification was based on 1011 yeast genomes that represent global yeast diversity (Figure 1A; Peter et al., 2018). Strikingly, rare variants contributed a disproportionate amount to phenotypic variation in both studies.

In one study Joseph Schacherer and co-workers at the University of Strasbourg – including Téo Fournier as first author – found that 16% of the GWAS results were rare alleles even when they made up just 4% of all the variants used in the experiments (Fournier et al., 2019). In the other study Joshua Bloom, Leonid Kruglyak and colleagues at UCLA estimated that over half of the observed growth variation can be explained by rare variants, even when they represented only 28% of the variants used (Bloom et al., 2019). The UCLA team also found that the rare variants detected in GWAS tend to have larger effect sizes than common variants, tend to reduce growth ability, and tend to have arisen more recently in evolutionary time.

These results join recent efforts exploring the effect of rare variants on complex traits. For human height it has been shown that rare variants have effect sizes ten times larger than common variants (Marouli et al., 2017), and that together they account for most of the missing heritability in this trait (Wainschtein et al., 2019). In parallel, it was estimated that at least a quarter of gene expression heritability in humans is accounted for by rare variants (Hernandez et al., 2019). The fact that in humans, as well as yeast, the contribution of rare variants to complex traits is now beyond doubt suggests that it may be the same in other species. However, addressing this question in organisms with larger genomes and not amenable to crossing schemes remains challenging. But rest assured, researchers will find a way.
